# Low dose radiotherapy combined with immune checkpoint inhibitors induces ferroptosis in lung cancer via the Nrf2/HO-1/GPX4 axis

**DOI:** 10.3389/fimmu.2025.1558814

**Published:** 2025-05-27

**Authors:** Jing Luo, Qiongjie Zhi, Dongxia Li, Yue Xu, Hui Zhu, Lujun Zhao, Guibing Ren, Jian Wang, Ningbo Liu

**Affiliations:** 1 Tianjin Medical University Cancer Institute and Hospital, National Clinical Research Center for Cancer, Tianjin, China; 2 Tianjin’s Clinical Research Center for Cancer, Tianjin, China; 3 Key Laboratory of Cancer Immunology and Biotherapy, Tianjin, China; 4 Department of Biotherapy, Tianjin Medical University Cancer Institute and Hospital, Tianjin, China; 5 Department of Respiratory Medicine, The Second People’s Hospital of Hefei, Hefei Hospital Affiliated to Anhui Medical University, Hefei, Anhui, China; 6 Department of Cardiology, Characteristic Medical Center of Chinese People’s Armed Police Force, Tianjin, China; 7 Department of Cancer Cell Biology, Tianjin Medical University Cancer Institute and Hospital, Tianjin, China; 8 Department of Radiation Oncology, Tianjin Medical University Cancer Institute and Hospital, Tianjin, China; 9 Department of Oncology, Characteristic Medical Center of Chinese People’s Armed Police Force, Tianjin, China; 10 Department of Immunology, Tianjin Medical University Cancer Institute and Hospital, Tianjin, China; 11 Department of Radiation Oncology, Hetian District People’s Hospital, Hetian, China

**Keywords:** low dose radiotherapy, immune checkpoint inhibitor, ferroptosis, Nrf2/HO-1/GPX4, chemoimmunotherapy-resistant

## Abstract

**Background:**

Immune checkpoint inhibitors (ICI) have revolutionized the therapeutic direction for lung cancer, yet their response rates remain unsatisfactory. Recently, the combination of ICI and low dose radiotherapy (LDR), a novel approach that effectively mobilizes innate and adaptive immunity, has gained interest among scientists. However, the underlying molecular mechanisms are not clearly elucidated.

**Methods:**

The *in vivo* anti-tumor effects of LDR and ICI were measured in murine tumor models. The immune response and alterations in the tumor microenvironment were measured using flow cytometry and enzyme-linked immunosorbent assay (ELISA). Cell viability and death were assessed using CCK-8 assays. Fluorescent probes and ELISA were used to assess ferroptosis induced by the combination therapy *in vitro* and *in vivo*. Western blotting and qPCR were performed to detect alterations in the Nrf2/HO-1/GPX4 pathway. Furthermore, a phase 1 clinical trial with a combined regimen of LDR and anti-PD-1 antibodies in patients with lung cancer was conducted.

**Results:**

The combined LDR and ICI regimen exhibited considerable anti-tumor effects in murine tumor models, promoting immune response and increasing the IFN-γ levels. *In vitro* data showed that LDR plus ICI induced ferroptosis in cancer cells by increasing reactive oxygen species and MDA levels, promoting Fe2^+^ accumulation, and suppressing GSH. Furthermore, ferroptosis induced by combination therapy was associated with suppression of the Nrf2/HO-1/GPX4 antioxidant axis. Importantly, a phase 1 clinical trial of the combination therapy showed promising efficacy in patients with lung cancer with chemoimmunotherapy resistance.

**Conclusion:**

This study demonstrated that LDR plus ICI induces ferroptosis through the Nrf2/HO-1/GPX4 pathway, resulting in a significant anti-tumor effect and providing a combinatorial strategy to overcome lung cancer. However, this combined strategy merits further clinical investigation.

## 1 Introduction

Lung cancer is the most lethal cancer worldwide and its burden remains significant (1). Over the past decade, great progress has been made in therapeutic options for lung cancer, particularly in immunotherapy. Immune checkpoint inhibitors (ICI) are the leading approach in cancer immunotherapy and have revolutionized the treatment of patients with cancer over the past decade. ICI have been incorporated into anti-cancer clinical practice owing to their broad bioactivity in various cancers, the stability of their response, and their efficacy even in chemotherapy-resistant malignancies (2). Treatment with anti-programmed cell death 1 (PD-1) and anti-PD-1 ligand 1 (PD-L1) antibodies has demonstrated substantial anti-tumor activity in multiple tumor types and has changed the treatment guidelines for lung cancer (3–5). PD-1 is a transmembrane protein that is widely expressed on the surface of immune cells, including activated T cells, B cells, monocytes, and can negatively regulate human immune responses by binding to its two ligands (PD-L1 and PD-L2) (6, 7). Anti-PD-1/PD-L1 inhibitors have become the standard therapy for various cancers and hold significant promise for tumor immunotherapy owing to their efficacy and precision (8). The PD-1/PD-L1 inhibitors nivolumab, pembrolizumab, cemiplimab, atezolizumab, avelumab, and durvalumab have received FDA approval since 2011 (9, 10). Numerous clinical studies have confirmed remarkable responses to PD-1/PD-L1 inhibitors across different cancer types. Patients with non-small cell lung cancer (NSCLC) (11), melanoma (12), urothelial carcinoma (13), head and neck squamous cell carcinoma (14), and renal cell carcinoma (15) have seen significant improvements in overall and progression-free survival when using PD-1/PD-L1 inhibitors. However, in clinical settings, medical management is often associated with treatment resistance, including the emergence of ICI resistance, which leads to failure or relapse (16, 17). Therefore, novel therapeutic models and combinations are required.

Radiation therapy (RT) is a vital therapeutic option worldwide for treating malignant tumors by inhibiting tumor cell growth and promoting cell death through reactive oxygen species (ROS) and DNA damage induction (18). We have gained a deeper understanding of the risks and benefits of RT; however, different regimens are still being explored to achieve the best treatment outcomes (19, 20). Different RT doses have been shown to exert different biological effects (21). Conventional and hypofractionated RT are used to treat malignant tumors because of their antiproliferative properties, breaking DNA double-strands and resulting in cell death (22). For human exposure, LDR is defined as low linear energy transfer radiation of up to 0.2 Gy or high linear energy transfer radiation of up to 0.05 Gy by the United Nations Scientific Committee on the Effects of Atomic Radiation (UNSCEAR) in 1986. The current practice is to maintain LDR doses between 0.5 and 2.5 Gy, with one or three exposures at various intervals, for a total dose of <10 Gy (23–26). Owing to the low single fraction (0.3–1.0 Gy) and total (3–6 Gy) doses, LDR causes little damage to DNA and has minimal effect on cells. It produces ROS in the irradiated area by electrolyzing water molecules within cells, thus regulating various cellular functions, and is widely used in the treatment of benign diseases such as arthritis (27, 28). Additionally, its significant anti-inflammatory effects (29) have been reported for decades, and its early application in clinical practice is attributed to its therapeutic potential against neurological disorders such as Alzheimer’s disease. Recently, LDR has been shown to play a key role in stimulating immune cell activation against tumors (30). As inflammation is often closely associated with immunity, regulation of the immune response is likely a central mechanism of LDR. An increasing number of studies have been conducted on LDR-mediated immunoregulation, and various immunoregulatory mechanisms have been identified (31). Since the emergence of immunotherapy, LDR has gained increasing attention, particularly in combination with radio-immunotherapy. It has been reported to enhance the efficacy of ICI, leading to significant progress in lung cancer radio-immunotherapy (32). However, the underlying mechanisms and specific combinations of LDR and ICI remain unclear. In the present study, we explored this gap and found that the LDR plus ICI combination can induce ferroptosis in lung cancer by involving tumor immunity and regulating metabolism.

Ferroptosis was first proposed by Stockwell in 2012 (33). Unlike necrosis, apoptosis, and autophagy, ferroptosis is an iron-dependent programmed mode of cell death (34). It is widely involved in physiological functions and tumor regulation, and is driven by iron-dependent phospholipid peroxidation. In this process, unsaturated fatty acids are peroxidized, which inhibits glutathione peroxidase 4 (GPX4), resulting in cell death. Ferroptosis is closely associated with disordered iron flow and ROS increments (35, 36), and is also linked with the pathological process and therapeutic prognosis of various diseases, including malignant tumors. Mounting evidence suggests its potential physiological functions in tumor immunity and metabolism (37, 38). Thus, ferroptosis regulation and development have become a major focus of cancer research and treatment. In the present study, we demonstrated that LDR combined with an ICI can induce ferroptosis in lung cancer, leading to tumor suppression and exerting a significant anti-tumor effect via activation of the Nrf2/HO-1/GPX4 axis. Our primary aim was to provide a theoretical and experimental basis for the clinical application of LDR combined with ICI in the treatment of lung cancer.

## 2 Materials and methods

### 2.1 *In vivo* studies

#### 2.1.1 Mice and treatment

**Figure 1:**
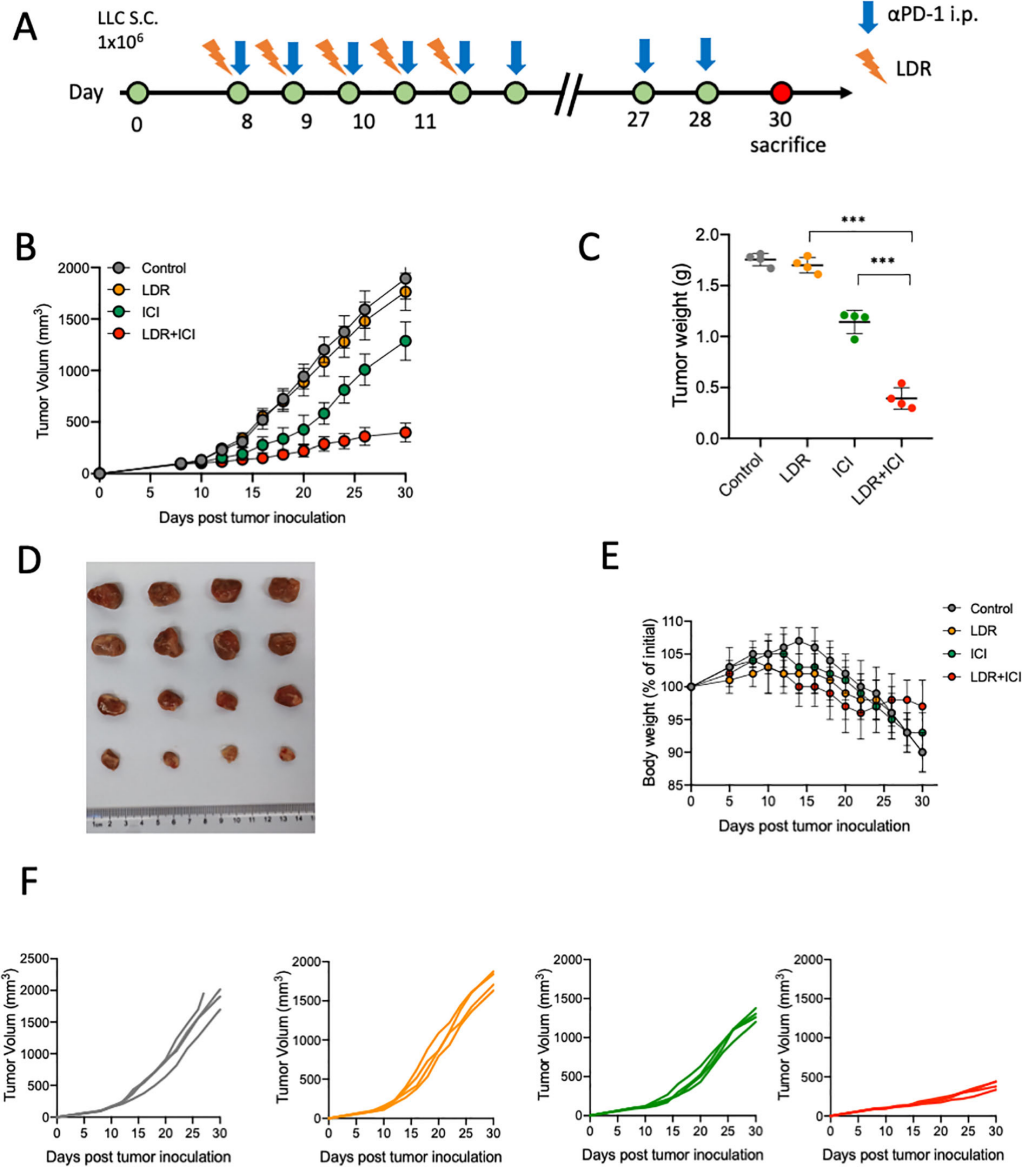
LDR combined with ICI had a significant anti-tumor effect in the LLC lung cancer model. **(A)** Workflow for the LLC lung cancer model and treatment with LDR combined with ICI. **(B)** Tumor volume, **(C)** body weight changes, **(D)** representative images of tumors, **(E)** tumor weights of mice bearing LLC tumors treated with LDR combined with ICI, as well as the combination therapy compared with control treatment (n = 4 mice per group). **(F)** Tumor growth curves of individual mice in different groups. LDR, low dose radiotherapy; αPD-1, anti-PD-1; i.p., intraperitoneal injection; ICI, immune checkpoint inhibitor. Data are presented as means ± SD. ***p < 0.01. Unpaired two-tailed Student’s t-test.

Six- to eight-week-old female wild-type C57BL/6 mice, weighing 18–22 grams, were purchased from SPF Biotechnology (Beijing, China). On day 0, 1 × 10^6^ LLC cells/100 μL phosphate-buffered saline (PBS) were injected into the right leg of the C57BL/6 mice. Tumor growth was monitored daily and measured every 1–2 days. Tumor volume was determined as length (mm) × width (mm^2^) × 0.5. When the tumor volume reached approximately 80–100 mm^3^, the mice were randomly divided into four treatment groups: Control, LDR treatment, ICI treatment, and combination treatment (LDR plus ICI). Each group contained at least four mice to ensure statistical significance. Based on preliminary experiments (**Supplementary Figure S1**), 1 Gy/fraction (f) was selected as the indicated LDR dose. Tumor-bearing mice received 6 MV-X ray, 5 Gy/5 fractions on days 8–13, with the irradiation localized to the tumor (right leg) and not the whole body. The mice were placed in a fixator and only the right leg was exposed. Vaseline (1.5 cm thick layer) was smeared on the tumor surface to avoid a dose build-up effect. A multileaf collimator in a medical accelerator (Elekta, Sweden) was used to create a radiation field, ensuring that only the exposed right thighs of the mice were in the field. For ICI treatment, tumor-bearing mice were treated with 200 μg αPD-1 monoclonal antibody daily. Mice in the control group were administered the IgG2a isotype. All treatment details are provided in **Figure 1A**. The mice were sacrificed when the tumor volume reached 2000 mm^3^. OT-I mice were purchased from Cyagen Biosciences (Santa Clara, CA). All mice experiments were conducted under pathogen-free conditions within a barrier facility, according to protocols approved by the Animal Ethical and Welfare Committee of Tianjin Medical University Cancer Institute and Hospital.

### 2.2 *In vitro* studies

For experiments conducted *in vitro* to induce ferroptosis, the LLC-OVA cells were treated differently. For the LDR group, cells received 0.5 Gy radiotherapy for one fraction. For the ICI treatment group, LLC-OVA were mixed with CD8^+^ T cells from OT-I mice (at a 1:1 ratio) in the presence of 50 μg/mL αPD-L1. For the LDR+ICI group, cells were exposed to both treatments.

### 2.3 Cells

LLC and LLC-OVA cells were cultured in a complete medium (DMED containing 10% fetal bovine serum and 1% penicillin-streptomycin). LLC cells were obtained from ATCC, and LLC-OVA cells were obtained from our laboratory. All the cell lines tested negative for mycoplasma contamination. CD8^+^ T cells were collected from the spleens of OT-I mice. After harvesting, the spleens were minced and strained through a 70-μm filter to obtain single-cell suspensions. CD8^+^ T cells were purified using magnetic beads (Miltenyi, USA), according to the manufacturer’s protocol.

### 2.4 Flow cytometry

Tumor tissues were harvested from mice, then minced and digested in a mixture of 160 μg/mL collagenase IV and 50 μg/mL DNase I in RPMI 1640 medium. The process was carried out at 37 °C for 30–40 min with agitation, and the digested tissues were strained through a 70 μm filter. For the *in vitro* experiments, LLC-OVA cells were treated using the same method. For the staining of single-cell suspensions, all incubations were performed on ice. Cells were first incubated with Zombie NIR (1/500), diluted in PBS for 30 min to distinguish between dead and living cells, washed twice, incubated with a mixture of antibodies in fluorescence-activated cell sorting buffer (2% fetal bovine serum in PBS) for 30 min, washed twice again, and suspended in staining buffer. Intracellular staining for chemokines was performed using Perm/Wash buffer, followed by a washing step, and suspension in the fluorescence-activated cell sorting buffer. Intranuclear staining of Foxp3 was performed using the Foxp3/Transcription Staining Buffer Set according to the manufacturer’s instructions. Data were collected using a BD LSRFortessa flow cytometer (BD Biosciences, San Jose, CA, USA) and analyzed using FlowJo version 9 (USA).

### 2.5 Western blotting

To assess the activation of the Nrf2/HO-1/GPX4 pathway, LLC-OVA cells were treated as described in the previous section and harvested from the different treatment groups. Total cell lysis buffer 1.1 × SDS containing a phosphatase inhibitor cocktail was used to generate whole-cell proteins. The quantity and quality of the proteins were confirmed using a NanoDrop system (DeNovix DS-11, Wilmington, DE, USA). Purified proteins were electrophoresed in 10% Tris-Glycine gels, transferred to polyvinylidene fluoride membranes blocked using 5% bovine serum albumin solution, and blotted with the corresponding primary antibodies, secondary antibodies, and horseradish peroxidase-conjugated IgG Ab for 2 h. The primary antibodies used were: anti-Nrf2 (1:2000, #ab62352, Abcam); anti-HO-1 (1:3000, #ab68477, Abcam); anti-GPX4 (1:5000, #ab125066, Abcam); anti-xCT (1:1000, #12691, Cell Signaling Technology); anti-β-actin (1:1000, #4967, Cell Signaling Technology). Membrane-bound complexes were detected using Image Studio version 5 (LI-COR Biosciences, Lincoln, NE, USA).

### 2.6 Enzyme-linked immunosorbent assay (ELISA)

For *in vivo* detection, blood samples were collected from the treated mice. Serum was isolated and diluted in PBS based on the range of ELISA detection. ELISA kits (Dakewe, China) were used to determine cytokine levels, and the assays were performed in accordance with the manufacturer’s instructions.

### 2.7 Real-time PCR

Tumors were isolated from the different treatment groups as described earlier. Total RNA was extracted from tumor tissues using TRIzol reagent according to the manufacturer’s instructions. Random primers and SuperScript III reverse transcriptase were used to synthesize cDNA, and quantitative RT-PCR was performed using the 2X SG Fast qPCR Master Mix (Low Rox) (Sangon, China), according to the manufacturer’s instructions.

### 2.8 Cell viability assay

Cell viability was assessed using a CCK-8 assay. LLC-OVA and ID8-OVA cells were seeded at a density of 2× 10^3^ cells/well in 96-well plates. After 24 h, once the cells had adhered properly, they were treated as described earlier. All treated cells were then incubated for 24 h at 37 °C with 5% CO_2_. The cell death inhibitors were used at the following concentrations: ferrostatin-1 (1 μM), Z-VAD-FMK (10 μM), and necrosulfonamide (1 μM). Subsequently, the medium was removed and CCK-8 solution (Solarbio, China) was added to each well to detect cell viability according to the manufacturer’s instructions.

### 2.9 Measurement of Fe2^+^ levels

Fe2^+^ levels were evaluated using a FerroOrange fluorescent probe (MedChemExpress). FerroOrange is a nonfluorescent cell-permeable dye that exhibits a fluorescent signal when bound to Fe2+ ions. The protocols were performed in accordance with the manufacturer’s instructions. Briefly, LLC-OVA cells were seeded in 12-well plates at 1 ×10^5^ cells/mL density and cultured until adherence. Subsequently, the cells were divided into the four treatment groups described earlier. After treatment, the cells were washed twice with a serum-free medium and treated with a working solution of 1 μmol/L FerroOrange fluorescent probe, prepared in serum-free medium. The cells were incubated for 30 min at 37°C in the dark, then washed twice with a serum-free cell culture solution. Images were captured using a confocal microscope (Olympus, Tokyo, Japan) at an excitation/emission wavelength of 542/572 nm. Finally, the fluorescence intensity of FerroOrange was quantified using the ImageJ software.

### 2.10 Measurement of MDA and GSH levels

MDA (Solarbio, China) and GSH (Solarbio) levels were assessed using specific assay kits. LLC-OVA cells were seeded in 6-well plates at a density of 5 ×10^4^ cells/well. Following cell adhesion, the cells were subjected to different treatments as described earlier. The MDA and GSH assays were performed according to the instructions provided by the respective kit manufacturers.

### 2.11 Detection of intracellular ROS levels

Intracellular ROS levels were evaluated using a DCFH-DA fluorescent probe (Beyotime, China). For *in vitro* ROS detection, LLC-OVA cells were cultured in 6-well plates at a density of 5 ×10^4^ cells/well. The cells underwent the treatments described earlier and were incubated for 6 h. Subsequently, they were washed with PBS and incubated with DCFH-DA (2 µL/well) for 30 min at 37 °C. The mean DCFH-DA level was measured using a BD LSR Fortessa flow cytometer (BD Biosciences, San Jose, CA, USA) and analyzed using FlowJo version 9 (USA). For *in vivo* ROS detection, tumor tissues collected from mice across the treatment groups were stored in liquid nitrogen. Frozen tissue sections (5 μm thick) were rewarmed and rinsed with PBS before use. A DHE kit (Solarbio, China) was used to detect tissue ROS levels based on the manufacturer’s instructions. Images were captured using a confocal microscope (Olympus, Tokyo, Japan), with an excitation/emission wavelength of 518/610 nm.

### 2.12 C11-BODIPY staining

Lipid peroxidation was determined using a C11-BODIPY 581/591 fluorescence probe (MedChemExpress, USA), according to the manufacturer’s instructions. Briefly, LLC-OVA cells were cultured in 6-well plates at a density of 5 ×10^4^ cells/well. Next, they were exposed to the different treatments described earlier and incubated for 6 h. After harvesting, the cells were washed and incubated with 3 μM of C11-BODIPY 581/591 working solution for 30 min at room temperature. Finally, images were captured using a confocal microscope (Olympus, Tokyo, Japan). BODIPY 581/591 C11 is emitted at 591 nm (reduced prototype), or redshifted to 510 nm (oxidized type). The excitation wavelengths were 581 nm (reduced prototype) and 500 nm (oxidized type).

### 2.13 Human studies

#### 2.13.1 Patients and design

The human study protocol was approved by the Ethics Committee of Tianjin Cancer Institute and Hospital (Ethical ID: E20240009, registration on ClinicalTrials.gov is ongoing), and was conducted in accordance with the Declaration of Helsinki. All the patients enrolled in the study provided written informed consent. These included six patients with pathologically confirmed NSCLC and all received at least four cycles of chemotherapy plus αPD-1 therapy. The enrolled patients received neoadjuvant paclitaxel 175 mg/m^2^ plus carboplatin (area under curve 5; 5 mg/mL per min) for squamous cell carcinoma, pemetrexed 500 mg/m^2^ plus carboplatin for adenocarcinoma, and intravenous sintilimab 200 mg on day 1, with 21 days in each cycle. After four cycles of chemoimmunotherapy, the patients were evaluated for progression disease (PD). For LDR, the targets and critical structures were delineated based on the CT images of the Philips Pinnacle8 treatment planning system (Philips Medical Systems, Cleveland, Oklahoma, USA) with the assistance of a radiation physicist. The radiation fields were defined according to the changes observed before and after systemic therapy. The gross tumor volume was defined as any visible tumor lesion on CT images (excluding lymph nodes identified from CT and/or PET scans). The planning gross tumor volume (PGTV) was defined according to Chen (39). The prescribed irradiation dose was 6 Gy to the PGTV, delivered as 1.2 Gy/f, QD. Considering the low total dose, the dose delivered to normal tissues was not limited.

### 2.14 Statistical analysis

GraphPad Prism 8.0 (GraphPad Software, Inc., La Jolla, CA) was used for statistical analysis, and results are expressed as the mean ± standard deviation (SD). Student’s t-test was applied to compare the differences between two groups, and p < 0.05 was considered statistically significant. *p < 0.05; **p < 0.01, ***p < 0.001.

## 3 Results

### 3.1 LDR combined with ICI has a significant anti-tumor effect and boosts IFN-γ levels

The efficacy of combination therapy using the mouse lung cancer tumor model, LLC cell lines were assessed. Mice bearing LLC murine lung cancer (sized 80–100 mm^3^) were treated with LDR and intraperitoneally with ICI (αPD-1). The treatment schedule is presented in **Figure 1A**. LDR monotherapy exhibited limited anti-tumor activity compared to the control and ICI monotherapy; however, combination therapy significantly delayed tumor growth (**Figures 1B–D**). Moreover, the mice that received the combination therapy experienced acceptable weight loss (**Figure 1E**). **Figure 1F** shows the tumor growth of each mouse in the different treatment groups. Significant inhibition of tumor growth *in vivo* was seen in the LDR plus ICI group compared with that in the monotherapy or control group.

**Figure 2:**
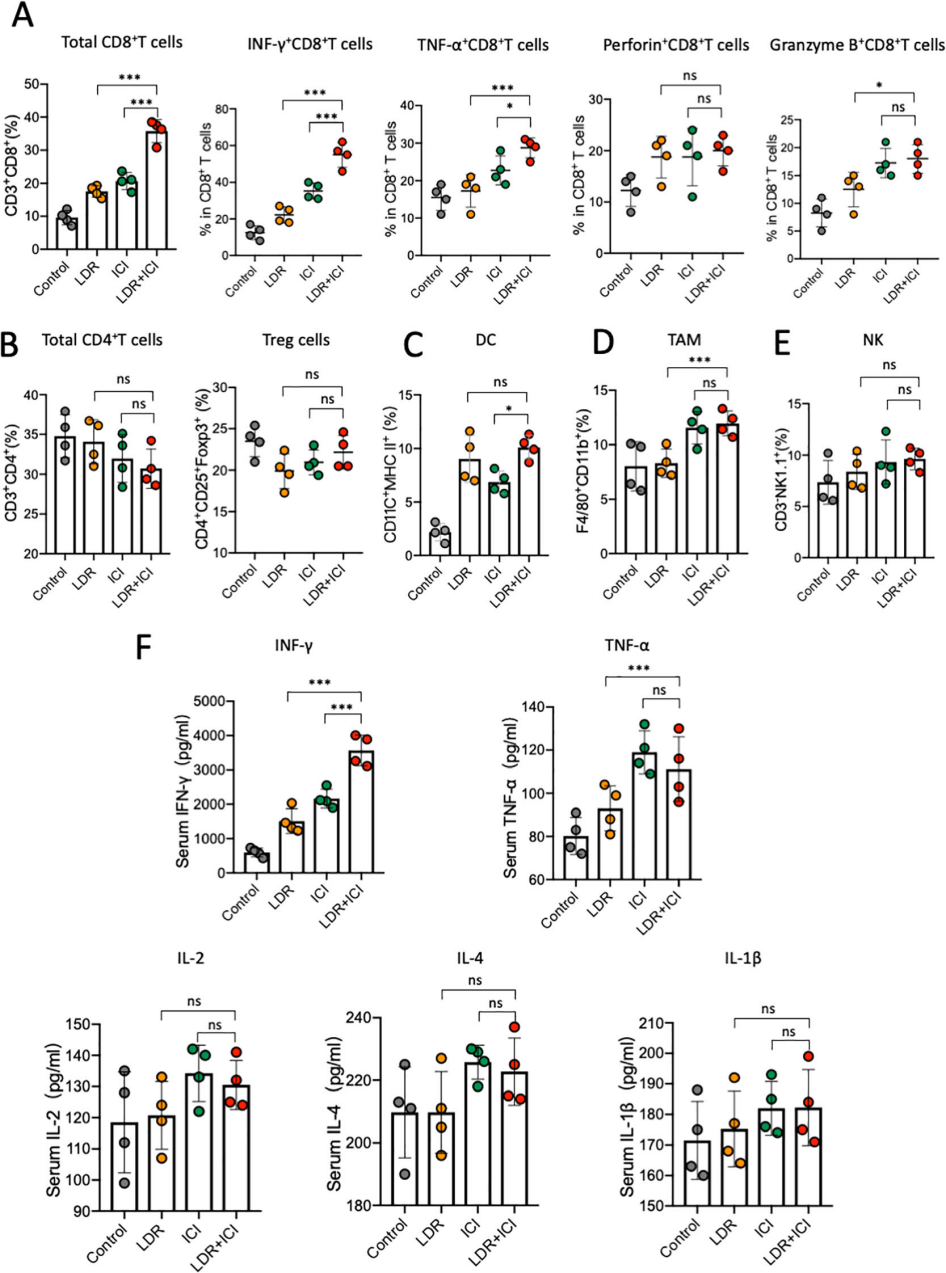
LDR combined with ICI increased IFN-γ levels in both the TME and blood. Quantification of tumor-infiltrating **(A)** CD8^+^ T cells, including IFNγ^+^ CD8^+^, TNFα^+^ CD8^+^, Perforin^+^ CD8^+^, and GranzymeB^+^ CD8^+^ TILs from mice across different treatment groups; **(B)** CD4^+^ T cells, including Tregs from mice across different treatment groups; **(C)** DC; **(D)** TAM; and **(E)** NK cells from mice across the different treatment groups. **(F)** ELISA of IFN-γ, TNF-α and important interleukins in the serum of mice in the different treatment groups. LDR, low dose radiotherapy; ICI, immune checkpoint inhibitor; DC, dendritic cell; TAM, tumor-associated macrophages; Treg, regulatory T cells; NK, natural killer; IL, interleukin. Data are presented as means ± SD. *p < 0.05; ***p < 0.01; ns, not significant. Unpaired two-tailed Student’s t-test.

The effects of combination therapy on the tumor microenvironment (TME) were also investigated. Tumor-infiltrating lymphocytes (TILs) in the TME were measured using flow cytometry. Compared to the control and monotherapy groups, LDR+ICI treatment increased the infiltration of CD8^+^ T cells (**Figure 2A**) and CD8^+^ cytotoxic lymphocytes (CTL) in tumors. Particularly, in comparison to other CTLs (TNF-α^+^ CD8^+^ TILs, Granzyme B^+^ CD8^+^ TILs, and Perforin^+^ CD8^+^ TILs), a marked increase in IFN-γ^+^ CD8^+^ TILs (**Figure 2A**) was detected in the combination group. Furthermore, other critical immune cells were monitored and observed differently. Compared to that in the monotherapy groups, no obvious increase in CD4^+^ T or regulatory T cells (Tregs; **Figure 2B**) was observed in the combination group. Similarly, the levels of tumor-infiltrating dendritic cells (DC), tumor-associated macrophages (TAM), and natural killer (NK) cells remained stable, with no significant differences between the monotherapy and combination groups (**Figures 2C–E**). Additionally, blood ELISA was performed to evaluate the secretion of important inflammatory cytokines. Only IFN-γ increased in the blood from mice in the combination group (**Figure 2F**), whereas other inflammatory cytokines such as TNF-α and interleukins remained stable across the different treatment groups. These results indicate that both LDR and ICI monotherapies have minor effects on TME, but LDR combined with ICI can significantly increase IFN-γ levels in both the TME and blood.

### 3.2 LDR combined with ICI induces ferroptosis in lung cancer cells by activating the Nrf2/HO-1/GPX4 axis

**Figure 3:**
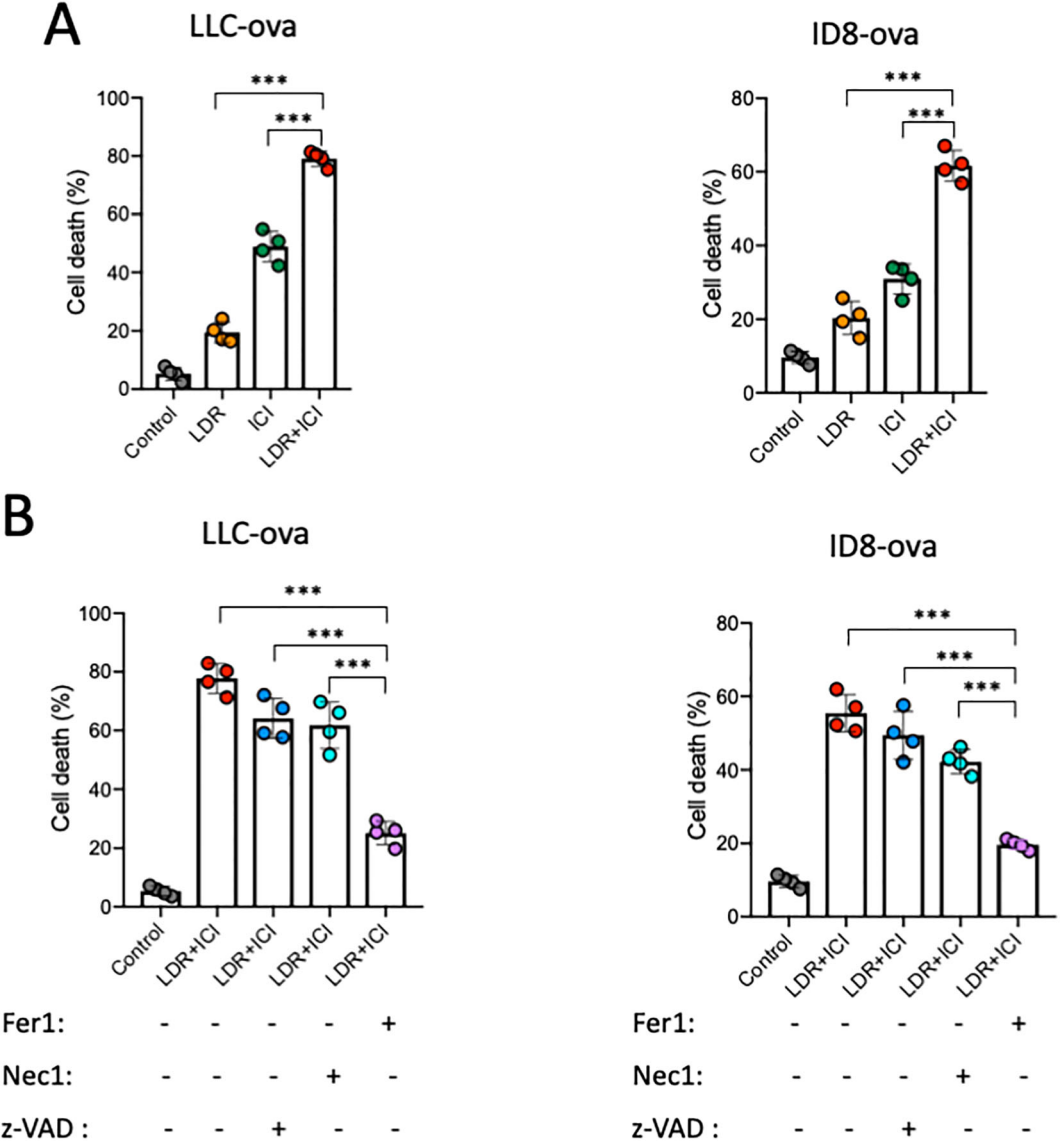
LDR combined with ICI induced cell death. **(A)** LDR combined with ICI induced cell death in LLC lung cancer and ID8 ovarian cancer cells. **(B)** Cell death induced by LDR combined with ICI in the absence and presence of ferrostatin-1 (1 μM), Z-VAD-FMK (10 μM), and necrosulfonamide (1 μM). LDR, low dose radiotherapy; ICI, immune checkpoint inhibitor; Fer1, ferrostatin-1; Z-VAD, Z-VAD-FMK; Nec1, necrosulfonamide. Data are presented as means ± SD. ***p < 0.01. Unpaired two-tailed Student’s t-test.

The effect of the combined therapy on the growth of lung cancer cells was assessed using a CCK-8 assay. The results revealed that LDR+ICI significantly inhibited the proliferation of LLC and ID8 cells. Following combined treatment for 24 h, more than half of the cells died, with approximately 60% and 80% of the LLC and ID8 cells dying, respectively (**Figure 3A**). To determine the type of cell death, a CCK-8 assay was performed using ferroptosis (ferrostatin-1), apoptosis (ZVAD-FMK), and necroptosis (necrosulfonamide) inhibitors. The results (**Figure 3B**) showed that cell death induced by LDR combined with ICI could be mostly reversed by a ferroptosis inhibitor, but not by inhibitors of apoptosis or necroptosis. Collectively, these findings demonstrate that LDR combined with ICI can induce ferroptosis in lung cancer cells.

**Figure 4:**
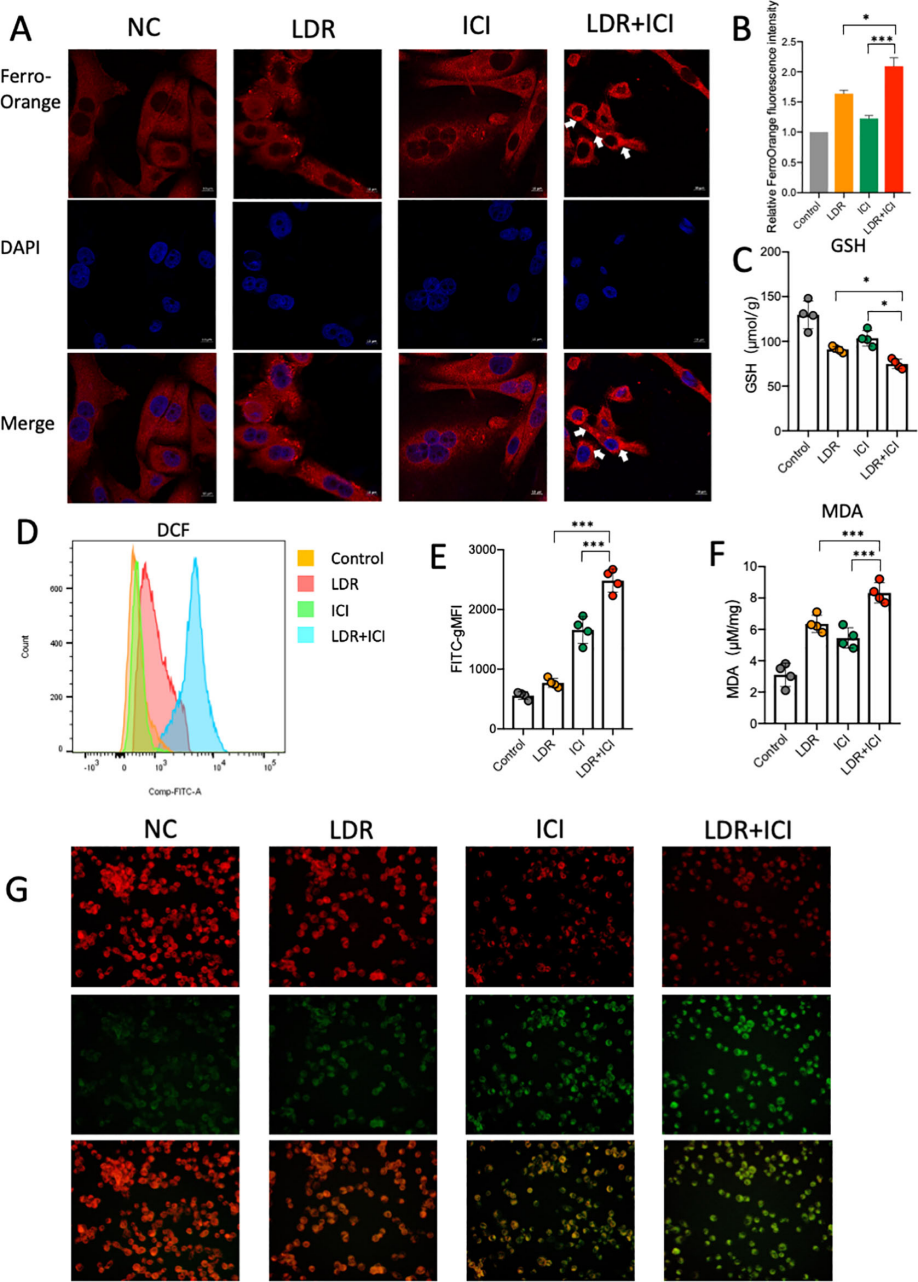
LDR combined with ICI induced ferroptosis and suppressed the Nrf2/HO-1/GPX4 pathway. Representative images of FerroOrange fluorescent **(A)** and fluorescent intensity **(B)** reflecting Fe2^+^ concentrations in LLC lung cancer cells in different treatment groups. **(C)** The GSH and MDA **(F)** levels of LLC lung cancer cells in different treatment groups. **(D)** Representative images of DCF fluorescent and fluorescent intensity **(E)** reflecting ROS levels of LLC lung cancer cells in different treatment groups. **(G)** Representative images of C11-BODIPY probe in LLC lung cancer cells across different treatment groups. Green fluorescence indicates oxidized lipid and red fluorescence indicates non-oxidized lipid. NC, normal control; LDR, low dose radiotherapy; ICI, immune checkpoint inhibitor; ROS, reactive oxygen species; gMFI, geometric mean fluorescence intensity. Data are presented as mean ± SD. *p < 0.05; ***p < 0.01. Unpaired two-tailed Student’s *t*-test.

We performed additional experiments to further investigate and confirm the potential mechanisms underlying ferroptosis induced by combination therapy. Ferroptosis is a special type of cell death associated with excessive lipid peroxidation, which requires iron. Iron exists in two oxidation states, ferrous [Fe^2^] and ferric [Fe^3+^], with Fe^2+^ accumulation representing the beginning of ferroptosis (40). A FerroOrange fluorescent probe was used to detect variations in Fe^2+^ concentrations in lung cancer cells (**Figure 4A**), and the fluorescence intensity was analyzed (**Figure 4B**). The results showed that both LDR and ICI can independently improve the intracellular Fe^2+^ concentration in lung cancer cells. However, this improvement was significantly greater in the case of combination treatment, with higher fluorescence intensity observed. In addition, we believe that the combined treatment resulted in a larger accumulation of Fe^2+^ in the cells. GSH and MDA levels were measured to quantify intracellular lipid peroxidation and reduced glutathione content. Data showed that in the combination group, GSH levels significantly decreased (**Figure 4C**), indicating that the cells had a weak antioxidant system (41). Based on the decreased GSH levels, we investigated ROS generation in the combination group. ROS serve as critical indicators of oxidative stress and are important activation signals for ferroptosis. DCFH-DA fluorescent staining was conducted to detect the intracellular ROS levels (**Figures 4D–E**). The results indicate that compared with the control and monotherapy groups, the LDR plus ICI group showed significantly elevated intracellular ROS levels. We then tested the levels of MDA, which reflects the degree of lipid peroxidation damage. Similar to ROS levels, MDA levels were elevated in the combination group, as shown in **Figure 4F**. Furthermore, the C11-BODIPY 581/591 probe was used to analyze the capacity of the combination therapy to induce lipid peroxidation. The shift of fluorescence from red to green represented lipid oxidation, indicating a decrease in red fluorescence and a strong increase in green fluorescence in the combined treatment group, when compared with the control and monotherapy groups. This suggests that the cells experienced an increase in lipid ROS levels and induced cell ferroptosis (**Figure 4G**). Collectively, the results indicate that LDR combined with ICI therapy can enhance oxidative stress and lipid peroxidation, thereby inducing ferroptosis in lung cancer cells.

**Figure 5:**
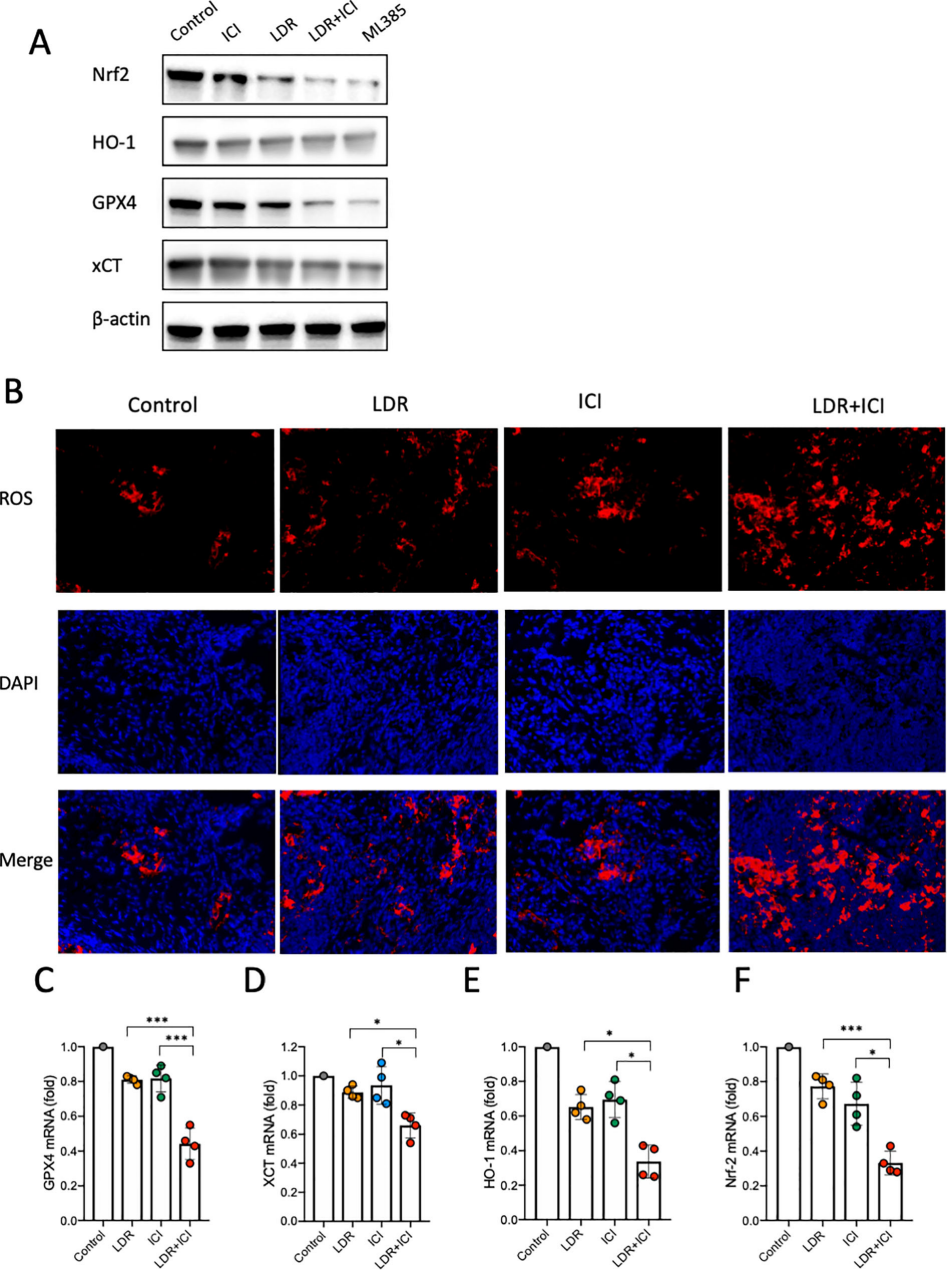
ROS levels and Nrf2/HO-1/GPX4 pathway analysis in mice across different treatment groups. **(A)** Western blot of Nrf2/HO-1/GPX4 activation in LLC lung cancer cells. **(B)** ROS levels in tumor tissues from mice. **(C-F)** Nrf2/HO-1/GPX4 pathway activation in tumor tissues from mice. LDR, low dose radiotherapy; ICI, immune checkpoint inhibitor; ROS, reactive oxygen species. Data are presented as mean ± SD. *p < 0.05; ***p < 0.01. Unpaired two-tailed Student’s *t*-test.

To elucidate the potential mechanism by which the combined treatment induces ferroptosis in lung cancer cells, we evaluated its effect on the Nrf2/HO-1/GPX4 antioxidant axis, considering that the decreased GSH is upstream of this pathway. ML-385, an Nrf2 inhibitor, was used as a positive control. As shown in **Figure 5A**, western blot data revealed a significant decrease in Nrf2, HO-1, and GPX4 expression levels in cells from the combination group compared to those from the monotherapy and control groups. Nrf2 is considered crucial for the cellular response to oxidative stress because it rapidly dissociates and translocates to the nucleus. This could activate the endogenous antioxidant factor HO-1, ultimately leading to activation of the Nrf2/HO-1 antioxidant defense system. Our data showed that the LDR combined with ICI treatment significantly suppressed the Nrf2/HO-1/GPX4 axis, indicating an impaired antioxidant system in lung cancer cells.

Our *in vitro* findings demonstrate that suppression of the Nrf2/HO-1/GPX4 axis is a possible mechanism by which the combined treatment induces ferroptosis in lung cancer cells. To test this hypothesis, we conducted *in vivo* experiments. Mice bearing LLC murine lung cancer were treated as described above (**Figure 1A**). Tumors were collected from each cohort after euthanasia. DHE staining was performed to detect ROS levels in the tumor tissues. The results showed that compared with the control and monotherapy groups, tumors from the LDR+ICI group showed a higher level of ROS generation (**Figure 5B**), which is consistent with our *in vitro* results. Furthermore, RNA extraction and subsequent qPCR experiments were performed to evaluate the Nrf2/HO-1/GPX4 pathway in tumor tissues. The results showed that the mRNA levels of Nrf2, HO-1, xCT and GPX4 decreased in mice treated with the combination therapy. This indicates that the combined treatment inhibited gene expression of the Nrf2/HO-1/GPX4 pathway and suppressed its activity (**Figures 5C–F**). These *in vivo* and *in vitro* data confirm that LDR combined with ICI therapy can induce ferroptosis in lung cancer cells by suppressing the Nrf2/HO-1/GPX4 axis.

### 3.3 LDR combined with ICI has a significant anti-tumor effect on patients with chemoimmunotherapy-resistant lung cancer

**Figure 6:**
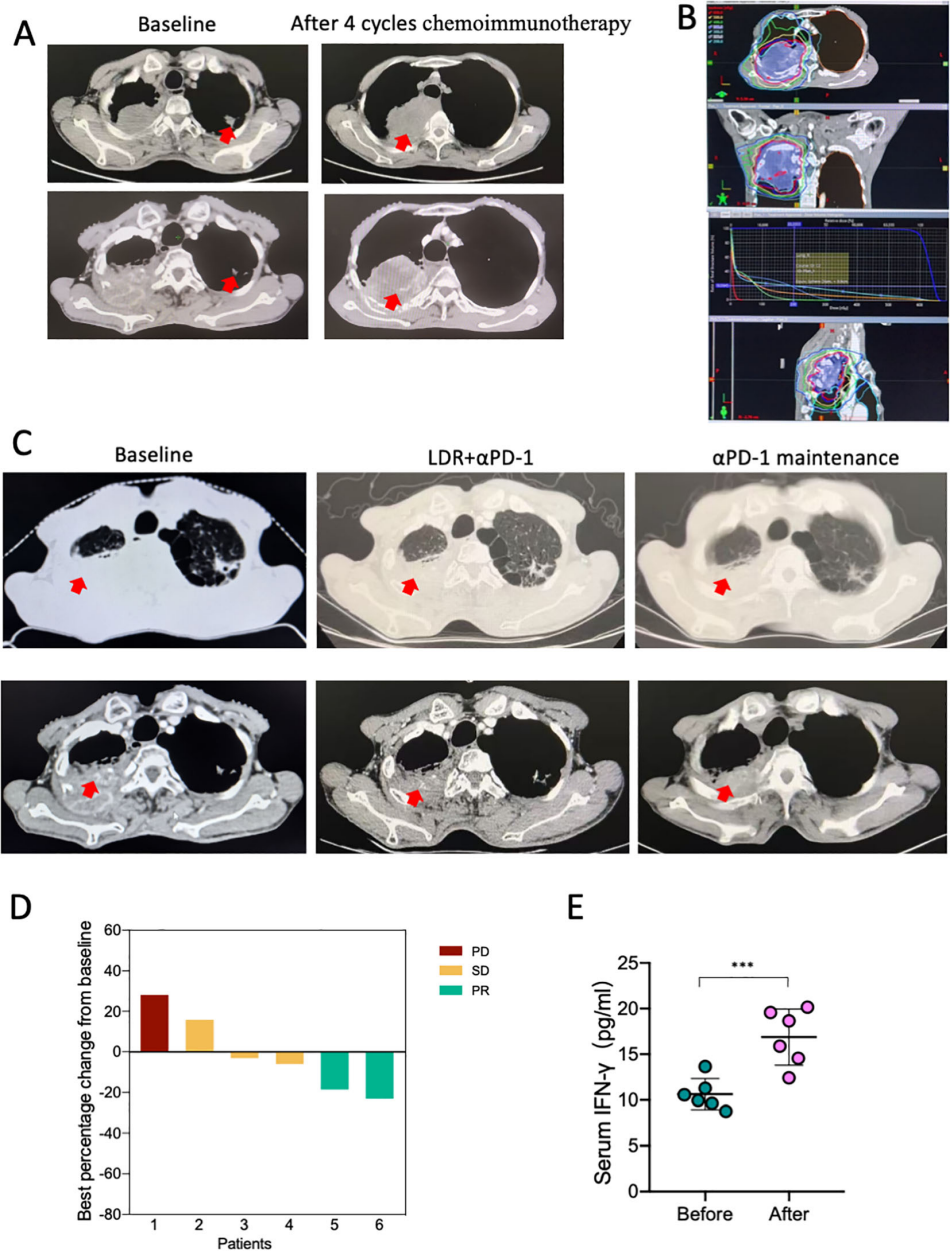
The combination of LDR and anti-PD-1 has an anti-tumor effect in patients with chemoimmunotherapy-resistant lung cancer. **(A)** CT images of a representative patient with NSCLC who underwent four cycles of chemoimmunotherapy and experienced PD. The patient received LDR plus αPD-1 therapy; the dose distribution plan is shown in (B). Following LDR and PD-1 treatment, the patient showed PR. The representative CT images are shown in **(C)**. **(D)** The best of response of the combination therapy from the base line. **(E)** Serum IFN-γ levels of the six patients before and after the combination therapy. NSCLC, non-small cell lung cancer; LDR, low dose radiotherapy; PD, progression disease; SD, stable disease; PR, partial response. Data are presented as mean ± SD. ***p < 0.01. Unpaired two-tailed Student’s *t*-test.

Based on the aforementioned data, we conducted a first-in-human, open-label, phase 1 clinical trial to assess the safety and preliminary efficacy of LDR combined with ICI. From March 1, 2024, to the present, approximately six patients with pathologically confirmed NSCLC were enrolled. All patients received at least four cycles of chemoimmunotherapy and were evaluated for PD; representative CT images are shown in **Figure 6A**. The patients then received LDR for one cycle. The dose distribution for the LDR treatment is provided in **Figure 6B**. At the data cutoff on December 1, 2024, all patients underwent one evaluable post-treatment tumor scan, showing a preliminary objective response rate of 33.3% (**Figure 6D**). A patient showing partial response (PR) after LDR therapy was also evaluated, and representative tumor images are shown in **Figure 6C**. Furthermore, we also monitored the IFN-γ levels in blood before and after treatment. An obvious increase in blood IFN-γ levels was observed in patients who received the combination therapy (**Figure 6E**). This result indicates that the combination therapy exerts anti-tumor immunity in humans, which is consistent with our results from lung cancer bearing mice.

## 4 Discussion

**Figure 7:**
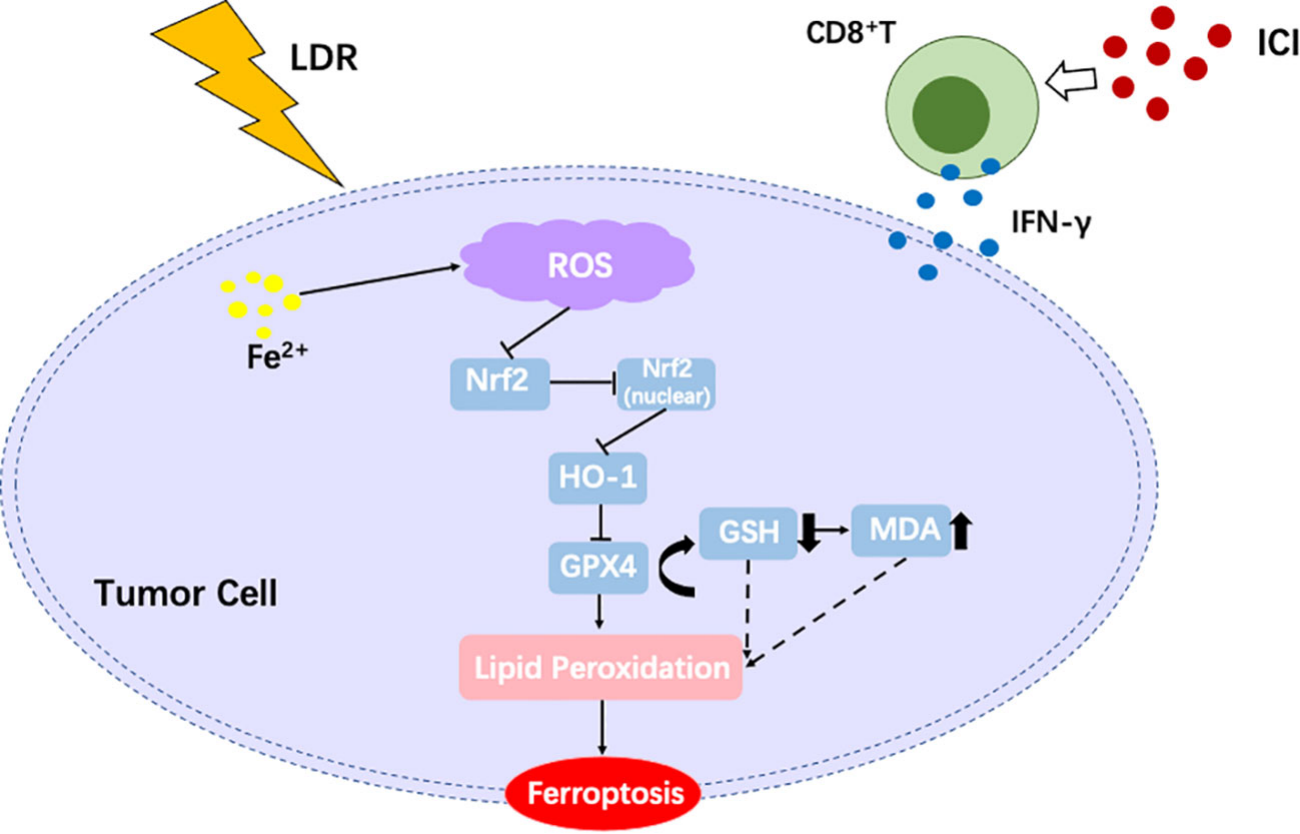
An overview of the present study.

For patients with lung cancer, including NSCLC and small cell lung cancer, significant progress has been made in improving survival over the last decade. In particular, the development of immunotherapies, especially those involving ICI, has changed the direction of clinical therapy (42, 43). For most patients with NSCLC, except those who harbor targetable oncogenes, anti-PD-1 or PD-L1 therapy is the first-line therapy (4, 42, 44, 45). While ICI have demonstrated remarkable efficacy in a subset of patients, challenges remain, particularly their limited clinical efficacy. The mean major pathological response rate to neoadjuvant anti-PD-1 or PD-L1 is approximately 32% (range, 18–63%) (46). Most patients become refractory to ICI therapy or develop resistance, which highlights the need for complementary therapeutic strategies (47). One such approach is LDR, which has gained attention for its ability to modulate the TME and enhance anti-tumor immunity (30). Interestingly, the mechanisms underlying ICI resistance, such as immunosuppressive TME and impaired T-cell infiltration, may be effectively targeted by LDR. By inducing immunogenic cell death and promoting antigen presentation, LDR can create a more favorable environment for ICI to exert their effects (48). In the present study, a novel radiotherapy schedule combined with ICI was attempted for the treatment of lung cancer, with encouraging results. Our study showed that LDR combined with ICI has a strong anti-tumor effect both in lung cancer models and patients, and the underlying mechanism involves inducing ferroptosis through suppression of the Nrf2/HO-1/GPX4 axis, an overview of this study has been provided in **Figure 7**.

Recently, LDR has gained interest in the scientific community for effective mobilization of innate and adaptive immunity. Based on several preclinical and clinical studies, LDR shows a promising ability to reprogram the TME, inducing the immune response and turning “cold tumor” to “warm tumor” (49). Studies have shown that the delivery of LDR combined with anti-CTLA4 increases the secretion of type I interferon by cancer cells, leading to the recruitment of DCs into the TME (50). Another study from the MD Anderson Cancer Center (51) combined LDR with anti-PD-1/PD-L1 therapy to stimulate immunological reprogramming in the TME of patients who had shown progress following anti-PD-1/PD-L1 therapy. Interestingly, this combination showed a higher overall response rate than traditional radiotherapy combined with anti-PD-1/PD-L1 therapy. However, the efficiency of LDR remains limited and has not yet translated into improvements in progression-free survival or overall survival. A phase 2 clinical trial (52) compared LDR and high-dose radiotherapy (HDR) combined with ICI in patients with NSCLC who have acquired resistance to ICI. Unfortunately, compared to HDR, LDR+ICI did not show an enhanced overall response or progression-free survival rate. Based on these results, we suggest that the LDR plus ICI therapy induces local reprogramming of the TME, but is insufficient to trigger a systemic immune response against tumors. LDR plays an important role in reversing innate or acquired ICI resistance; however, the optimal radiotherapy schedule and timing to stimulate immune-mediated tumor responses remain a challenge. In our preliminary clinical data, 6 Gy/5f combined with αPD-1 in patients with acquired chemoimmunotherapy resistance achieved a good objective response rate (33.3%). Compared with the data from the MD Anderson Cancer Center, the objective response rate was 26% for LDR+ICI and 13% for HDR+ICI (51). This may be because our patients were more susceptible to ICI treatment. Additionally, the survival outcomes of our patients were not available, and long-term observations are ongoing. Further research is required to explore the optimal LDR–ICI combination plan and clarify the underlying mechanism.

In our study, the Nrf2/HO-1/GPX4 pathway emerged as a key pathway for LDR plus ICI to induce ferroptosis, consequently leading to cancer cell death. Nrf2/HO-1/GPX4 is an important antioxidant pathway that is widely involved in metabolic activities (53). The activation of Nrf2/HO-1/GPX4 can provide resistance to oxidative stress, protect mitochondrial integrity, prevent mitochondrial dysfunction, and enhance the effectiveness of cell defense (54, 55). The accumulation of oxidative stress, such as ROS, in cells can result in ferroptosis (56). Our study revealed that the combination therapy can induce an increase in ROS levels while suppressing the Nrf2/HO-1/GPX4 antioxidant pathway, leading to ferroptosis in lung cancer cells.

Another potential mechanism is indicated by the elevated INF-γ levels. A study reported that CD8^+^ T cells activated by immunotherapy increased the level of ferroptosis-specific lipid peroxidation in tumor cells, thus inducing ferroptosis and enhancing the antitumor effect (57). In this mechanism, the increased levels of IFN-γ secreted from activated CD8^+^ T cells could suppress SLC3A2 and SLC7A11 (two important subunits of the glutamate-cystine antiporter system xc-) and restrain tumor cell cystine uptake, thus leading to lipid peroxidation and ferroptosis in tumor cells (57). Another investigation found that IFN-γ secreted from activated CD8^+^ T cells can induce ferroptosis by regulating lipid metabolism (58). IFN-γ can stimulate ACSL4, a type of lipid metabolizing enzyme that belongs to the long-chain acyl-CoA synthetase (ACSL) family. This family converts long-chain fatty acids into their corresponding acyl-CoAs and plays an important role in phospholipid remodeling (59). ACSL4 stimulated by IFN-γ can change the lipid pattern of tumor cells, thereby enhancing the incorporation of arachidonic acid (AA) into C16 and C18 acyl chain-containing phospholipids. Palmitoleic acid and oleic acid, two common C16 and C18 fatty acids in the blood, promote ACSL4-dependent tumor ferroptosis induced by IFN-γ plus AA (59). These results confirm that high levels of IFN-γ from activated CD8^+^ T cells can induce ferroptosis.

In our study, activated CD8^+^ T cells as well as IFN-γ^+^ cytotoxic T lymphocytes were observed in the TME of the LDR plus ICI therapy group. Moreover, high levels of IFN-γ were also found in blood. These results indicate that the LDR plus ICI therapy exerts anti-tumor immunity and enhances the activation of CD8^+^ T, leading to high levels of IFN-γ in both the TME and blood. We speculate that the increased IFN-γ promotes tumor ferroptosis, induced by the suppression of the Nrf2/HO-1/GPX4 axis.

Immunotherapy resistance remains a significant challenge in the treatment of lung cancer, limiting the efficacy of ICI such as anti-PD-1/PD-L1 antibodies. Resistance contributes to the inability of ICI to sustain effective anti-tumor immunity, leading to disease progression in a substantial proportion of patients (47, 60). LDR has emerged as a promising strategy for overcoming these challenges. Specifically, it induces immunogenic cell death by releasing tumor-associated antigens and damage-associated molecular patterns that stimulate dendritic cell maturation and T cell activation. This process can potentially reverse the immunosuppressive TME and restore sensitivity to ICI (48). The combination of LDR and ICI also holds promise in addressing the heterogeneity of immune resistance. According to our study, LDR combined with ICI induces ferroptosis in lung cancer cells, resulting in an antitumor effect. More importantly, we found that the combination therapy increases intracellular ROS levels and suppresses Nrf2/HO-1/GPX4 activation. Furthermore, LDR plus ICI proved beneficial in patients with lung cancer in a clinical trial. Therefore, LDR combined with ICI is a promising therapeutic strategy for the treatment of patients with lung cancer.

In conclusion, integration of LDR with ICI is a promising strategy for overcoming immunotherapy resistance in lung cancer. By modulating the TME and inducing ferroptosis, LDR addresses multiple resistance mechanisms and synergizes with ICI to achieve durable anti-tumor responses. Future clinical trials should explore the optimal dosing and sequencing of LDR and ICI to maximize their therapeutic efficacy and translate these findings into clinical practice.

However, the present study has some limitations. Both increase in IFN-γ and downregulation of the Nrf2/HO-1/GPX4 axis may be responsible for ferroptosis induced by the LDR plus ICI therapy. The exact mechanism by which IFN-γ promotes ferroptosis could not be clarified. Further investigation is required to fill this gap. Furthermore, although the phase I clinical trial revealed that LDR combined with ICI has a positive anti-tumor effect on patients with chemotherapy resistance, ferroptosis and its potential mechanism were not fully explored. Considering that the clinical trial is ongoing, these mechanisms must be investigated.

## Data Availability

The raw data supporting the conclusions of this article will be made available by the authors, without undue reservation.
