# Supplementary material for: Low dose radiotherapy combined with immune checkpoint inhibitors induces ferroptosis in lung cancer via the Nrf2/HO-1/GPX4 axis
Source: Front Immunol. 2025 May 27;16:1558814. doi: 10.3389/fimmu.2025.1558814 (PMC12178237; doi:10.3389/fimmu.2025.1558814)
Supplement: Supplementary file 1 [file DataSheet1.docx]

Figure S1


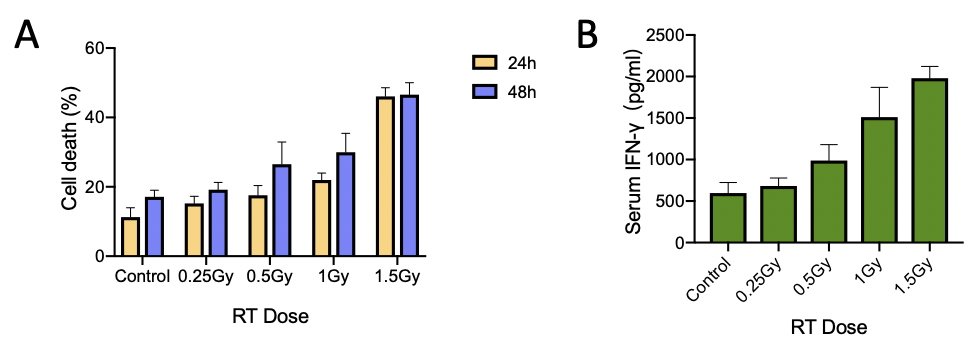


Figure S1: (A) Different LDR dose on the influences of LLC cell death. (B) Different LDR dose on the influences of the INF-γ production in LLC-bearing mice. RT, radiotherapy; Gy, gray; h, hour. Data are presented as mean ± SD.
